# *In-silico* proteomic analysis of the role of IL-4 and IL-10 in IVD degeneration: Protein-protein interaction networks for candidate prioritisation

**DOI:** 10.1016/j.csbj.2025.04.015

**Published:** 2025-04-14

**Authors:** Paola Bermudez-Lekerika, Francesco Gualdi, Christine L. Le Maitre, Janet Piñero, Baldomero Oliva, Benjamin Gantenbein

**Affiliations:** a Tissue Engineering for Orthopaedics & Mechanobiology, Bone & Joint Program, Department for BioMedical Research (DBMR), Faculty of Medicine, University of Bern, Murtenstrasse 35, Bern CH-3008, Switzerland; b Graduate School for Cellular and Biomedical Sciences (GCB), University of Bern, Mittelstrasse 43, Bern CH-3012, Switzerland; c Group of Integrative Biomedical Informatics, Hospital del Mar Research Institute, Barcelona 08003, Spain; d Istituto Dalle Molle di Studi sull'Intelligenza Artificiale (IDSIA), USI/SUPSI, Lugano, Switzerland; e Division of Clinical Sciences, School of Medicine and Population Health, University of Sheffield, Sheffield S10 2RN, United Kingdom; f Group of Structural Bioinformatics, Department of Medicine and Life Sciences, Universitat Pompeu Fabra, Barcelona 08003, Spain; g Department of Orthopaedic Surgery and Traumatology, Inselspital, Bern University Hospital, Faculty of Medicine, University of Bern, Freiburgstrasse 3, Bern CH-3010, Switzerland; h Medinformatics Solutions SL, Barcelona 08007, Spain

**Keywords:** Low back pain, Intervertebral disc degeneration, PPI network, Proteomics, Candidate prioritisation, Prioritisation algorithm

## Abstract

Protein-protein interaction (PPI) networks provide a static map of functional protein interactions, which when combined with algorithms, can prioritize key protein candidates which experimental studies cannot capture. This study, aimed to construct knowledge-based nucleus pulposus (NP)-specific PPI networks which could be deployed to investigate complex protein interactions in human NP cells and tissues following IL-4 and IL-10 stimulation. NP-specific PPI networks were developed based on mass spectrometry (MS) and secretome datasets from human NP cells. These networks were validated using in vitro and ex vivo experimental data sets. Genes Underlying Inheritance Linked Disorders (GUILD) genome-wide network-based prioritization framework was employed for protein candidate prediction under no treatment baseline and IL-4, IL-10 and IL-1β single or combined stimulating scenarios. These secretome-based in vitro PPI networks were able to reproduce the no-treatment candidate prioritization baseline. Whereby within NP cells from discs isolated due to traumatic injury biglycan was identified whilst in degenerate samples decorin was highlighted. Furthermore, experimentally observed IL-4 pleiotropic behaviour was predicted by IL-1 receptor-like 1 prioritization. PPI network-based IL-4 and IL-10 conditions offered novel insights of potential candidates, including collagen IV and fibroblast growth factor intracellular binding protein (FIBP) as key candidates within IL-4 activation pathways, whereas urocortin 3 and neural growth factor were identified following IL-10 stimulation. Additionally, MS based PPI network propagation offered a more extensive, module-based structure networks with lower edge degree and biological variability. Overall, multiple proteomic experimental approaches are required to successfully validate in-silico prediction models to understand the complex interactions between the plethora of proteins involved in IVD degeneration.

## Graphical Abstract

**Figure:**
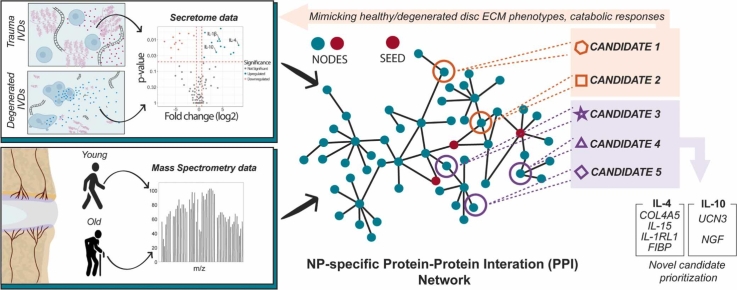
PPI network offer an intuitive and static approach to understanding the underlying complex interactions between biological components that experimental studies cannot capture. This study aimed to build, validate, and employ NP-specific PPI networks to further interpret large-scale data previously published. As a result, PPI network propagation methods could behave similar to in-vitro or ex-vivo experiments and reliably prioritize potential protein candidates involved in the IVD ECM structure, cell signalling and catabolic responses

## 1 Introduction

Low back pain (LBP) is a highly prevalent condition and the leading cause of disability worldwide. Globally, 619 million LBP cases were estimated in 2020, and is the leading cause for years lived with disability (YLD) [1]. The societal and economic burden of LBP stems from working-age people suffering this condition together with health-care costs and loss of workdays [2], [3], [4]. While LBP is multifactorial, with genetic, biological, and psychosocial components, IVD degeneration has been associated with approximately 40 % of chronic LBP cases [5]. The IVD consists of an avascular proteoglycan-rich nucleus pulposus (NP), which is laterally constrained by the peripheral fibre-reinforced annulus fibrosus (AF), situated between two cartilage endplates (CEP) of adjacent vertebral bodies, which are responsible for nutrient, oxygen, and waste transport [6]. During degeneration, different structural and biochemical alterations occur in the IVD, including loss of proteoglycans resulting in decreased osmotic pressure and hydration [7]. This causes a reduction in disc height, an accumulation of structural defects, and nerve ingrowth, promoting discogenic pain [8]. In the last decades, a wide range of cytokines have been applied *in-vitro* or *ex-vivo* to IVD cells to investigate the degenerative cascade. Tumour necrosis factor (TNF) or interleukin-1 β (IL-1β) have been described as key regulators of the catabolic processes during IVD degeneration [9], [10], [11], [12]. In contrast, GDF5 and GDF6 factors have been reported as key anabolic mediators in the disc [13], [14], [15]. Whilst other regulators are less well understood, for example a recent IVD-specific in-silico regulatory model proposed IL-4 and IL-10 as potential anabolic factors in the IVD [16]. The underlying mechanisms which regulate a plethora of cytokines, chemokines and regulators in the IVD remain poorly understood.

Computational methodologies have been used to aid in the exploration and elucidation of the biology of complex and multifactorial diseases [17], [18]. Computational methods can analyse large-scale and high dimensional biological data to identify possible biomarkers associated with disease, physiological states or response to treatment which would not be possible with for example individual in vitro stimulation studies [19], [20]. Omics technologies are an attractive option to assess temporal, spatial and cellular features from the IVD environment [21]. However, the complexity of analysing this data is multiple, not only due to its substantial volume, but also the selection of a mathematical framework that can efficiently represent this complexity in a scalable manner. As such within the manuscript a collaborative effort, which involved contributions from biology, mathematics, and computer science, aimed to implement tissue-specific PPI networks to prioritize key protein candidates within the IVD.

In biological systems, entities do not exist in isolation but are integral components of larger, interconnected systems. Thus, network in-silico approaches have emerged as valuable resources for investigating complex diseases [22]. Briefly, a network or graph (*G*) can be defined as a pair *G* = (*V*, *E*), where *V* represents a set of elements known as nodes (or vertices), and *E* denotes a set of paired nodes, with its elements referred to as edges (or links). Thus, entities can be depicted as nodes and relationships as edges connecting these nodes. Edges in a network can be considered directed or undirected depending on the presence of a specific direction. Every node in a particular network has a degree (i.e. the number of edges that are incident to a specific node), indicating its level of connectivity within the network. Therefore, nodes with a high degree are often considered essential hubs or central connectivity points, while a lower degrees may serve as peripheral or isolated components. An example of a biological network is a protein-protein interaction (PPI) network in which, nodes are proteins, and an edge exists between two proteins if they interact. PPI networks can be constructed with the aid of large repositories [23], [24] which collect experimentally validated protein interactions. Thus, network-based approaches can exhibit promising outcomes across numerous biomedical domains [25]. In addition, the analysis of PPI networks can identify pleiotropy [26], frequently found in proteins considered central nodes which contain more interactors compared to non-pleiotropic proteins [27], [28].

In the IVD context, in the last decade molecular in-silico investigations of IVD degeneration have relied on the implementation of network analysis mainly on PPI networks [29], [30]. This study aimed to build, validate and employ NP-specific PPI networks to further interpret large-scale data from previously published spatiotemporal proteomics atlas of human IVDs [31] or secretome analysis [32] and utilise these networks to prioritise key protein candidates under different scenarios.

## 2 Methods

### 2.1 Data acquisition

To generate substrate networks to run the *in-silico* experiments, publicly available repositories and previously reported IVD related *in-vitro* and *ex-vivo* studies were integrated. Firstly, data sets from mass spectrometry (MS) experiments performed on human cadaveric young (16 years old) and old (59 years old) nucleus pulposus (NP) tissue were utilized to select key intracellular and extracellular proteins from an NP-specific spatial proteome under different ageing scenarios [31]. However, key secreted cytokines and chemokines previously reported on the IVD [9] were not measured, and thus the network lacked the secretome component. Thus, a second dataset whereby a large Luminex panel had been utilise to profile secretome proteomics from *in-vitro* and *ex-vivo* human NP cells and tissue [32] was utilized to enhance the network with secreted proteins following specific stimuli. Furthermore, IL-4, IL-10 and IL-1β stimulated secretome profiles were employed to build different PPI networks and thus evaluate the potential influence or pleiotropism of these interleukins on different phenotypic scenarios. Finally, the interactions between the selected proteins were established according to Human Integrated Protein – Protein Interaction Reference (HIPPIE, version 2,3) [33] repository. HIPPIE offers a comprehensive collection of reliable and meaningful human based PPI. This dataset contains more than 270,000 confidences scored and annotated PPIs, integrating different types of experimental information and thus suitable for protein-protein interactome network development.

### 2.2 Network generation

An initial interactome was obtained with HIPPIE [33] containing interactions between human proteins. For each experimental case (Table 1) a specific network was generated by filtering the initial interactome according to the proteins of interest. MS-based Dipper [31]data set was utilized to filter the initial interactome for all the proteins present in nucleus pulposus (NP) tissue from one young and one old patient [31]. In this way, an IVD-specific PPI network was built followed by the addition of the proteins of interest and their neighbours according to each condition (Table 1).

**Table 1:** Experimental cases according to utilized data set, phenotype and condition.

Data set	Phenotype	Condition
**Secretome proteomics**	Human NP cells from trauma IVDs – based PPI Network (Trauma PPI Network)	*No treatment baseline*
		*IL−4 condition*
		*IL−1β condition*
	Human NP cells from degenerated IVDs – based PPI Network (Degenerated PPI Network)	*No treatment baseline*
		*IL−4 condition*
		*IL−10 condition*
		*IL−1β condition*
		*IL−1β + IL−4 condition*
		*IL−1β + IL−10 condition*
	Human NP explants – based PPI Network (Explant PPI Network)	*No treatment baseline*
		*IL−4 condition*
		*IL−1β condition*
		*IL−10 condition*
		*IL−1β + IL−4 condition*
**Mass Spectrometry proteomics**	Young PPI Network	*No treatment baseline*
		*IL−4 condition*
		*IL−10 condition*
	Old PPI Network	*No treatment baseline*
		*IL−4 condition*
		*IL−10 condition*

Furthermore, secretome-based data sets were divided in *in-vitro* NP cells from patients undergoing disc removal due to trauma (trauma PPI network) and discectomy due to degenerated discs (degenerated PPI network); and *ex-vivo* explants which were obtained from patients with degenerate discs (explant PPI network) dataset. These datasets were then utilized to filter the initial interactome according to different treatments (1 ng/ml IL-1 β, 10 ng/ml IL-4 or 10 ng/ml IL-10 either alone or IL-1β in combination with either IL-4 or IL-10)(Table 1) followed by an enrichment step in which all the nodes in the network were connected through their shortest path (i.e. the minimum number of connected proteins between 2 nodes). In this way we obtained a fully connected interactome.

### 2.3 GUILD algorithm and candidate categorization

GUILD [34] is a genome-wide network-based prioritization framework that includes multiple algorithms for network propagation exploiting the PPI interaction network to predict proteins involved in a specific phenotype. This method is built upon the guilt-by-association principle i.e. that proteins which are associated or interact would share similar pathways [35]. This study utilized NetScore algorithm with default parameters. The algorithm took as an input an interactome of PPI and a list of proteins ‘named seeds’ starting with a score higher than 0. The score, which represented the relevance of these proteins in a specific biological context, was propagated by a message passing through the edges of the interactome, generating a ranked list of prioritised proteins. Finally, top ranked candidates were selected according to the size of the network. For the secretome-based PPI network, which contained 782 nodes, the top 5 % and top 2 % prioritised candidates were selected, obtaining 4–37 unique proteins under each condition. For MS-based PPI network containing over 1400 nodes, the top 2 % and top 0.25 % were selected obtaining 3–290 unique proteins. To enable direct comparison the same top 2 % was evaluated in both approaches, as well as a similar number of prioritised candidates. Furthermore, UniprotKB protein sequence knowledgebase [36] was utilized for protein candidate categorization.

### 2.4 Seed selection

A seed is defined as protein of interest that forms a specific biological context [37]. Seeds can come from various sources, such as differentially expressed proteins identified in proteomics or transcriptomics experiments or prior knowledge of disease-associated proteins from the literature. These seeds are then mapped onto a PPI network, which serves as prior biological knowledge and the starting point for the signal in the network propagation algorithm. In this study, different combinations of seeds were selected for each theoretical experiment. Within MS-based networks, 25 seeds previously described in the nucleus pulposus (NP) [38] were selected and followed by the addition of IL-4 and IL-10 nodes (as conditions) in both young and old scenarios (Table 2). In the next step, each seed was scored as 1 and the rest of the nodes as 0, while IL-4 and IL-10 were additionally scored as 1 under their respective conditions, to simulate their addition.

**Table 2:** Secretome and MS-based PPI network description according to different experimental conditions.

Data	Condition		Nodes	Seeds	Diameter
**Secretome proteomics**	**Trauma PPI Network**	*No treatment Baseline*	782	SERPINE1	5
		*IL−4 condition*	782	SERPINE1, IL−4	5
		*IL−1β condition*	782	SERPINE1, IL−4, IL−1β	5
	**Degenerated PPI Network**	*No treatment Baseline*	782	MMP2	5
		*IL−4 condition*	782	MMP2, IL−4	5
		*IL−10 condition*	782	MMP2, IL−10	5
		*IL−1β condition*	782	MMP2, IL−1β	5
		*IL−1β + IL−4 condition*	782	MMP2, IL−1β, IL−4	5
		*IL−1β + IL−10 condition*	782	MMP2, IL−1β, IL−10	5
	**Explants PPI Network**	*No treatment Baseline*	782	MMP2	5
		*IL−4 conditions*	782	MMP2, IL−4	5
		*IL−1β condition*	782	MMP2, IL−1β	5
		*IL−10 condition*	782	MMP2, IL−10	5
		*IL−1β + IL−4 condition*	782	MMP2, IL−1β, IL−4	5
**Mass Spectrometry proteomics**	**Young PPI Network**	*No treatment Baseline*	14646	CX3CL1, IL−6ST, IL−1β, IL−6, CXCL8, IL−18, IL16, GDF5, OSM, CXCL2, GDF6, IL−17D, CCL3, IL−20, TGFB1, CCL5, PMP2, LIF, CCL7, TNF, IGF1, CCL2, VEGFA, CXCL3, CXCL1	8
		*IL−4 conditions*	14646	CX3CL1, IL−6ST, IL−1β, IL−6, CXCL8, IL−18, IL16, GDF5, OSM, CXCL2, GDF6, IL−17D, CCL3, IL−20, TGFB1, CCL5, PMP2, LIF, CCL7, TNF, IGF1, CCL2, VEGFA, CXCL3, CXCL1, IL−4	8
		*IL−10 condition*	14646	CX3CL1, IL−6ST, IL−1β, IL−6, CXCL8, IL−18, IL16, GDF5, OSM, CXCL2, GDF6, IL−17D, CCL3, IL−20, TGFB1, CCL5, PMP2, LIF, CCL7, TNF, IGF1, CCL2, VEGFA, CXCL3, CXCL1, IL−10	8
	**Old PPI Network**	*No treatment Baseline*	14407	CX3CL1, IL−6ST, IL−1β, IL−6, CXCL8, IL−18, IL16, GDF5, OSM, CXCL2, GDF6, IL−17D, CCL3, IL−20, TGFB1, CCL5, PMP2, LIF, CCL7, TNF, IGF1, CCL2, VEGFA, CXCL3, CXCL1	8
		***IL−4 conditions***	14407	CX3CL1, IL−6ST, IL−1β, IL−6, CXCL8, IL−18, IL16, GDF5, OSM, CXCL2, GDF6, IL−17D, CCL3, IL−20, TGFB1, CCL5, PMP2, LIF, CCL7, TNF, IGF1, CCL2, VEGFA, CXCL3, CXCL1, IL−4	8
		***IL−10 condition***	14646	CX3CL1, IL−6ST, IL−1β, IL−6, CXCL8, IL−18, IL16, GDF5, OSM, CXCL2, GDF6, IL−17D, CCL3, IL−20, TGFB1, CCL5, PMP2, LIF, CCL7, TNF, IGF1, CCL2, VEGFA, CXCL3, CXCL1, IL−10	8

According to secretome-based networks, the protein with the highest concentration was selected as a seed, considering SERPINE1 as a seed for the trauma PPI network; and MMP2 for degenerated and explant networks, followed by IL-4, IL-10 and IL-1β nodes (as conditions). However, the scoring system followed a different strategy. In a first step, the expression levels of three biological replicates were averaged and normalized by the minimum and maximum protein expression scaling calculated as:

$$X_{\mathrm{sc}}=\;\frac{X-X_{\min}}{X_{\max}-X_{\min}}$$

Where $X_{\mathrm{sc}}$ is the scaled value, X the original secreted protein-protein expression value and $X_{\min}$ and $X_{\max}$ are respectively the lower and the higher secreted protein expression values. Finally, for every condition the respective secretome profile was utilized for building the network and the stimulating protein (IL-4, IL-10 and/or IL-1) were forced to obtain the same values as the most expressed protein (i.e. 1), mimicking and emphasizing *in-vitro* or *ex-vivo* stimulation.

### 2.5 Network analysis

For every baseline interactome different metrics were calculated as follows:

$$(\mathrm{AD})\mathrm{Average degree}=\;\frac{2\times E}{N}$$

Where E is the number of edges and N is the number of nodes of the network. The average degree gives insight into how interconnected the nodes in a network are, which is useful in network analysis across various fields such as social networks, biological networks, and communication networks.

$$\left(C_{a}\right)\mathrm{Average clustering coefficient}=\;\frac{1}{N}\sum_{i=1}^{N}C_{i}$$

Where $C_{i}$is the clustering coefficient calculated for the N number of nodes of the network. The average clustering coefficient is often used in analysing how much a network tends to form interconnected communities [39].

$$(D)\mathrm{Network density}=\;\frac{2|E|}{|N|(\left|N\right|-1)}$$

Where E is the number of edges of the network and N is the number of nodes. Thus is a metrics on how many connections are present in the network in relation to the maximum possible number of connections and gives information on how tightly the nodes are in the network connected.

$$\left(Q\right)\mathrm{Modularity}=\;\frac{1}{2E}\sum_{\mathrm{ij}}\left[A_{\mathrm{ij}}-\frac{k_{i}k_{j}}{2E}\right]\delta (c_{i},c_{j})$$

Where $A_{\mathrm{ij}}$ is the adjacency matrix of the graph, $k_{i}k_{j}$ are the degrees of the nodes i and j respectively, E is the total number of edges in the graph $c_{i},c_{j}$ are the communities to which nodes i and j belong and $\delta (c_{i},c_{j})$ is 1 if i and j are in the same community and 0 otherwise. Modularity is a metric that measures how well it is divided into communities or modules, maximizing iteratively by Louvain algorithm [40] to obtain the ideal module separation. A module (or community) is a group of nodes that are more densely connected to each other than to the rest of the network. Modularity helps quantify the quality of this division by comparing the density of links inside communities versus the density of links between different communities.

All the calculations and the plots were produced using Python 3.11.5 and R [41] coding language, and RStudio (R version 4.4.1, R studio Team, 2020, RStudio, PBC, Boston, MA) software including ggven [42] and ggplot2 [43] packages.

## 3 Results

### 3.1 Secretome and MS-based interactomes produced different size networks

Secretome-based and MS-based interactomes were described according to the number of nodes, diameter and seeds. Expectedly, MS-based interactomes contained a higher number of nodes (from 14,407 to 14,646 nodes) compared to secretome-based interactomes (782 nodes) (Table 2). Furthermore, interactome diameter consisting of links between nodes was higher in MS-based interactomes, containing eight links between 16 nodes (Table 2). According to the secretome-based PPI network scoring system, the most expressed protein was employed to normalise the protein expression of the rest of the nodes, selecting the SERPINE-1 node as a seed for the trauma PPI network baseline. In contrast, MMP2 protein was the most expressed node in degenerated and explant PPI networks, and thus, selected as seeds (Table 2). MS-based interactomes, however, integrated previously described cellular mediators such as cytokines and chemokines in the nucleus pulposus (NP) [38] into their interactome selecting them as seeds (Table 2).CX3CL1: Chemokine (C-X3-C motif) ligand 1; IL-6ST: Interleukin 6 signal transducer; IL-1β: Interleukin 1 beta; IL-6: Interleukin 6; CXCL8: Chemokine (C-X-C motif) ligand 8 (also known as IL-8); IL-18: Interleukin 18; IL-16: Interleukin 16; GDF5: Growth differentiation factor 5; OSM: Oncostatin M; CXCL2: Chemokine (C-X-C motif) ligand 2; GDF6: Growth differentiation factor 6; IL-17D: Interleukin 17D; CCL3: Chemokine (C-C motif) ligand 3 (also known as MIP-1α); IL-20: Interleukin 20; TGFB1: Transforming growth factor beta 1; CCL5: Chemokine (C-C motif) ligand 5 (also known as RANTES); PMP2: Peripheral myelin protein 2; LIF: Leukemia inhibitory factor; CCL7: Chemokine (C-C motif) ligand 7; TNF: Tumor necrosis factor; IGF1: Insulin-like growth factor 1; CCL2: Chemokine (C-C motif) ligand 2 (also known as MCP-1); VEGFA: Vascular endothelial growth factor A; CXCL3: Chemokine (C-X-C motif) ligand 3; CXCL1: Chemokine (C-X-C motif) ligand 1 (also known as GROα); MMP2: Matrix metallopeptidase 2 (also known as gelatinase A); IL-4: Interleukin 4; SERPINE1: Serpin peptidase inhibitor, clade E, member 1 (also known as PAI-1); IL-10: Interleukin 10. Node: Proteins represented in a network; Seed: protein of interest that forms a specific biological context; Diameter: longest path between two nodes. For secretome-based networks, the protein with the highest concentration was selected as a seed, considering SERPINE1 as a seed for trauma PPI networks while MMP2 for degenerated and explant networks, followed by IL-4, IL-10 and IL-1β nodes (as conditions). For MS-based networks, 25 seeds previously described in the nucleus pulposus were selected and followed by IL-4 and IL-10 nodes as conditions.

### 3.2 MS-based PPI candidate networks exhibited increased number of nodes, modularity and decreased network density

**Fig. 1:**
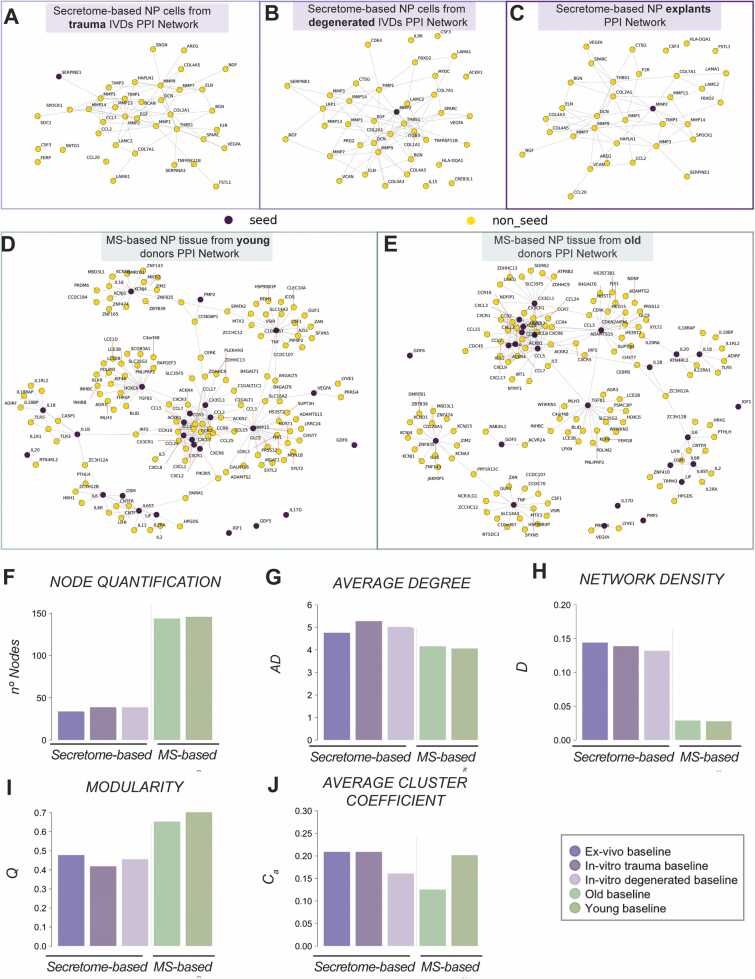
The top 5 % candidate networks from secretome-based *in-vitro* NP cells from (A) trauma human IVDs, (B) degenerated human IVDs, and (C) *ex-vivo* explants PPI network. Top 1 % candidate network baselines from MS-based human NP tissue from (D) young and (E) old patients’ PPI network. Evaluation and comparison of (F) Average degree, (G) Network density, (H) Modularity and (I) Global Cluster Coefficient metrics between MS and secretome-based PPI network baselines.

The top 5 % and 1 % ranked protein nodes were selected for secretome and MS-based no-treatment conditions respectively to evaluate prioritised candidate interconnectivity (Figs. 1A, 1B, 1C, 1D, and 1E). Generally, secretome-based candidate baseline networks had a smaller number of nodes (≈ 40 nodes) compared to MS-based candidate baseline (≈ 145 nodes) even though the selected top percentage were five times higher (Fig. 1F). Interestingly, the average degree showed a slightly decreased value in MS-based candidate networks and a strongly reduced network density (Figs. 1G and 1H). In contrast, the modularity was higher in the MS-based baseline networks (Fig. 1I). Furthermore, the overall level of clustering determined by the average clustering coefficient was similar between the baselines, while the smaller value was reported in the MS-based baseline for young human donors followed by a secretome-based *in-vitro* baseline from NP cells from degenerated IVDs (Fig. 1J).

### 3.3 *In-vitro* and *ex-vivo* phenotypic scenarios exhibit specific candidate prioritization

**Fig. 2:**
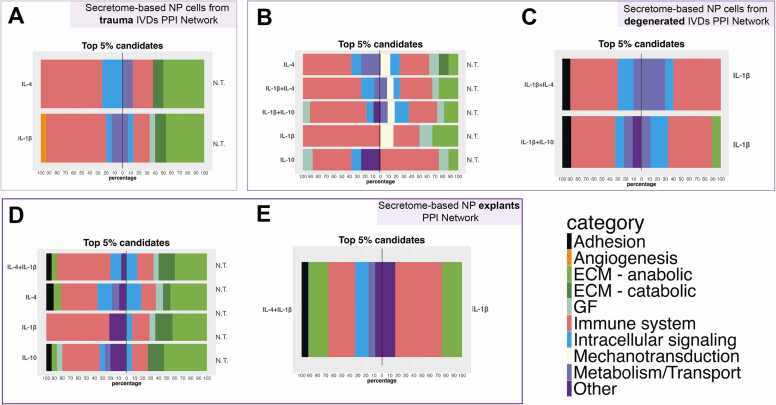
Stacked bar chat of top 5 % protein candidate classification that were uniquely prioritised in each single or combined 10 ng/ml IL-4, IL-10 or 1 ng/ml IL-1β treatments (left) compared to (A) no treated NP cells from trauma discs, (B) no treated NP cells from degenerated discs, (C) IL-1β treated NP cells from degenerated discs, (D) no treated NP explants and (E) IL-1β treated NP cells top 5 % candidate lists (right) obtained in secretome-based PPI networks. N.T.= No treatment.

The top 5 % of prioritised proteins were selected from *in-vitro* (trauma and degenerated) and *ex-vivo* secretome-based PPI networks (Table S1) and compared between different conditions, obtaining shared and unique candidates (Table 3). Initially, the trauma PPI network baseline (no stimulation) revealed primarily ECM anabolic and catabolic and immune system-related proteins (Fig. 2A). Additionally, the trauma PPI network under both IL-4 and IL-1β conditions exhibited an immune system component, prioritizing IL-6, IL-7, IL-11, IL-13 and IL-15 and IL-1RL1. Furthermore, different chemokines were highlighted between conditions, CXCL-16 being unique for the IL-4 scenario, while CXCL1, CXCL12, and resistin (RETN) were unique for the IL-1β condition (Table 3). On the other hand, prioritised candidates from degenerated PPI network no treatment baselines shared an enhanced immune system-related profile, including antimicrobial proteoglycan 2 (PRG2), colony stimulant factor 3 (CSF-3), atypical chemokine receptor 1 (ACR1) as well as the presence of endothelial growth factor (EGF) and integrin β3 (ITGβ3) (Fig. 2B). Similarly, unique protein candidates listed for different conditions revealed the presence of IL-13, IL-7, IL-2, asialoglycoprotein Receptor 2 (ASGR2), and fibroblast growth factor FGF1 intracellular binding protein (FIBP) for IL-4 containing conditions; IL-6, IL-11, CCL2, CCL20 and oncostatin M (OSM) for IL-1β group and IL-22, amphiregulin (AREG), X-C chemokine ligand 2 (XCL2) and urocortin 3 (UCN3) for IL-10 containing conditions (Table 3). Interestingly, unique candidates from IL-1β combined treatments compared to a single IL-1β baseline revealed an adhesion-related protein named myocilin (MYOC) (Fig. 2C, Table S1). Finally, candidate lists from the explant PPI network baseline exhibited similar protein prioritisation to that seen in the trauma PPI network baseline, including ECM, adhesion, and immune system-related proteins (Fig. 2D). Interestingly, MYOC and TNFRSF11B proteins were prioritised in IL-4 and IL-10 scenarios conditions: IL-13; cyclic AMP-responsive element-binding protein 3-like protein 1 (CREB3L1); ASGR2; RETN; Yip1 domain family member 6 (YPF6); jagunal homolog 1 (JGN1) and sydecan-1 (SDC1) for IL-4 containing conditions, and IL-1α; VWD domain containing protein (VWDE); brevican (BCAN); interleukin 1 receptor accessory protein (IL-1RAP); CCL21; cholinergic receptor nicotinic Alpha 5 subunit (CHRNA5); C-X-C motif chemokine ligand 10 (CXCL10); C-C motif chemokine ligand 7 (CCL7); were unique for IL-1β groups (Fig. 2D). Additionally, multiple categories, including adhesion, anabolic, immune system, intracellular, metabolism, and transport, were listed exclusively for combined IL-4 and IL-1β conditions compared to single IL-1β baseline prioritisation (Table S1) (Fig. 2E).

**Table 3:** Top 5 % prioritised candidates in secretome-based PPI networks.

Condition		Prioritised categories	Unique Proteins
**Trauma PPI Network**	***No treatment Baseline***	Principally ECM related anabolic and catabolic proteins	SERPINA3, FSTL1, THBS1, LAMC2, CSF3, SNTG1
	***IL−4 condition***	Immune system related proteins and intracellular signalling proteins	LRP5, CXCL16, IL7, IL13, IL15, RETN, FIBP, **IL−4**
	***IL−1β condition***	Immune system related proteins, intracellular signalling and angiogenic proteins	ATP8B2, CD53, OSM, RETN CASP1, PPP1R12C, LIF*, CTPS2, CXCL12, COL4A3 CXCL1, IL11**,** IL6, IL1RL1*, **IL−1β**
**Degenerated PPI Network**	***No treatment Baseline***	Principally Immune system related proteins	ACKR1, PRG2, SERPINE1, CSF3, EGF, ITGβ3
	***IL−4 containing conditions***	Immune system proteins	IL7*, IL2, IL13, ASGR2, FIBP, **IL−4**
	***IL−1β condition***	Immune system proteins	CCL20, OSM, CCL2, IL6 IL11, **IL−1β**
	***IL−10 containing conditions***	Immune system proteins and growth factor receptors	IL22 AREG, XCL2, UCN3, **IL−10**
**Explants PPI Network**	***No treatment Baseline***	Mainly ECM related anabolic and catabolic proteins	FSTL1, HAPLN1, CTSG, AREG, COL2A1, FBXO2, CSF3, THBS1, LAMC2, VCAN, F2R
	***IL−4 containing conditions***	Principally immune system related proteins	YIPF6, SDC1, JAGN1, IL13, MYOC, CREB3L1, ASGR2, TNFRSF11B, RETN, FIBP, **IL−4**
	***IL−1β condition***	Immune system proteins	IL1A, VWDE, XCL2, BCAN, IL1RAP, CCL21, CHRNA5 CXCL10, CCL7, IL11, **IL−1β**
	***IL−10 condition***	Principally immune system related proteins	SRGN, IL11, FKRP, XCL2, EGF, TNFRSF11B, NMU, MYOC, TIMP3, SNTG1, JAGN1, RETN, YIPF6, UCN3 **IL−10**

ACKR1: Atypical Chemokine Receptor 1; AREG: Amphiregulin; ASGR2: Asialoglycoprotein Receptor 2; ATP8B2: ATPase Phospholipid Transporting 8B2; BCAN: Brevican; CASP1: Caspase 1; CCL2: C-C Motif Chemokine Ligand 2; CCL20: C-C Motif Chemokine Ligand 20; CCL21: C-C Motif Chemokine Ligand 21; CCL7: C-C Motif Chemokine Ligand 7; COL2A1: Collagen Type II Alpha 1; COL4A3: Collagen Type IV Alpha 3; CREB3L1: cAMP Responsive Element Binding Protein 3-Like 1; CSF3: Colony Stimulating Factor 3; CXCL1: C-X-C Motif Chemokine Ligand 1; CXCL10: C-X-C Motif Chemokine Ligand 10; CXCL12: C-X-C Motif Chemokine Ligand 12; CXCL16: C-X-C Motif Chemokine Ligand 16; EGF: Epidermal Growth Factor; F2R: Coagulation Factor II Receptor; FIBP: FGF1-Binding Protein; FKRP: Fukutin Related Protein; FBXO2: F-Box Protein 2; FSTL1: Follistatin-Like 1; HAPLN1: Hyaluronan and Proteoglycan Link Protein 1; IL10: Interleukin 10; IL11: Interleukin 11; IL1A: Interleukin 1 Alpha; IL1β: Interleukin 1 Beta; IL1RL1: Interleukin 1 Receptor-Like 1; IL2: Interleukin 2; IL4: Interleukin 4; IL6: Interleukin 6; IL7: Interleukin 7; IL13: Interleukin 13; IL15: Interleukin 15; IL22: Interleukin 22; JAGN1: Jagunal Homolog 1; KLK2: Kallikrein-Related Peptidase 2; LAMC2: Laminin Subunit Gamma 2; LIF: Leukemia Inhibitory Factor; LRP5: Low-Density Lipoprotein Receptor-Related Protein 5; NMU: Neuromedin U; OSM: Oncostatin M; PRG2: Proteoglycan 2; PPP1R12C: Protein Phosphatase 1 Regulatory Subunit 12 C; RETN: Resistin; SERPINA3: Serpin Family A Member 3; SERPINE1: Serpin Family E Member 1; SNTG1: Syntrophin Gamma 1; THBS1: Thrombospondin-1; TIMP3: Metalloproteinase Inhibitor 3; UCN3: Urocortin 3; VWDE: Von Willebrand factor D and EGF domains; VCAN: Versican; YIPF6: Yip1 Domain Family Member 6. * Significantly upregulated proteins observed in the experimental data. **In bold:** selected treatments in the experimental data and selected seed in the PPI networks.

### 3.4 Trauma phenotypic NP cell networks can mimic IL-1β mediated catabolic response

**Fig. 3:**
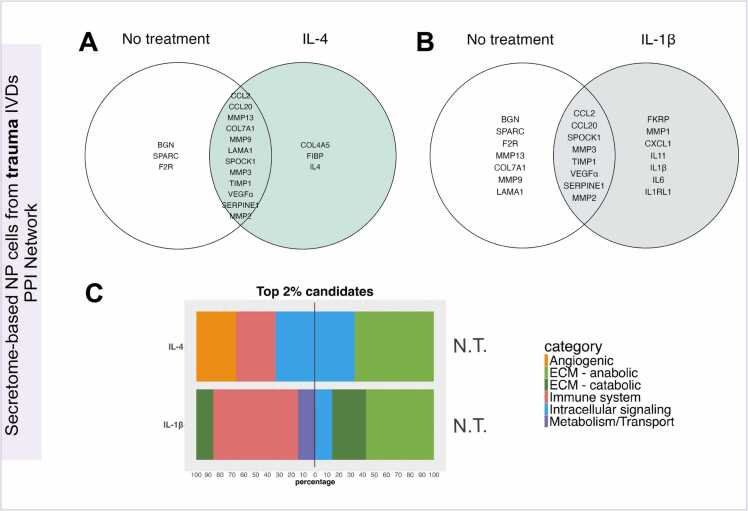
Top 2 % protein candidates uniquely prioritised in 10 ng/ml IL-4 and 1 ng/ml IL1β treatments in in-vitro NP cell from trauma discs secretome-based PPI networks. Venn’s diagram shows the top 2 % unique protein candidates under (A) IL-4 condition or (B) IL-1β condition compared to no treatment group. (C) Stacked bar chart of top 2 % protein candidate classification that were uniquely prioritised in IL-4 or IL1β treatments (left) compared to no treated candidate list (right). N.T. = No treatment.

The relevance of each protein candidate was then ranked by narrowing down the prioritised candidate list and highlighting key protein candidates in each network. Following this, only the top 2 % of listed candidates were analysed on *in-vitro* trauma and degenerated secretome-based network models (Table S2). As a result, COL4A5 and FIBP proteins were identified as most relevant nodes in trauma PPI network under IL-4 condition. At the same time, biglycan (BGN), osteonectin (SPARC) and proteinase-activated receptor 1 (F2R) were exclusively identified in the no treatment control baseline (Fig. 3A). For the IL-1β scenario, IL-11, IL-6, IL-1RL1, CXCL1, metalloproteinase (MMP) 1 and ribitol 5-phosphate transferase (FKRP) were rated as top 2 % most relevant candidates whereas BGN, SPARCK, F2R, MMP4, COL7A1, MMP1 and laminin A1 (LAMA1) were uniquely selected for no treatment baseline (Fig. 3). According to the candidate’s classification, no treatment baselines remained similar, containing ECM-related candidates such as anabolic proteins and intracellular signalling mediators. In contrast, both IL-1β and IL-4 conditions exhibited a strong immune system related component, more pronounced in the IL-1β condition. Furthermore, ranked candidates for IL-4 condition were less numerous and included angiogenesis and intracellular signalling candidates, while catabolic ECM proteins, metabolism and transport proteins were prioritised in IL-1β scenario (Fig. 3C).

### 3.5 *In-vitro* IL-4 and IL-10 conditions enhance immune system related protein prioritization on degenerated phenotypic networks

**Fig. 4:**
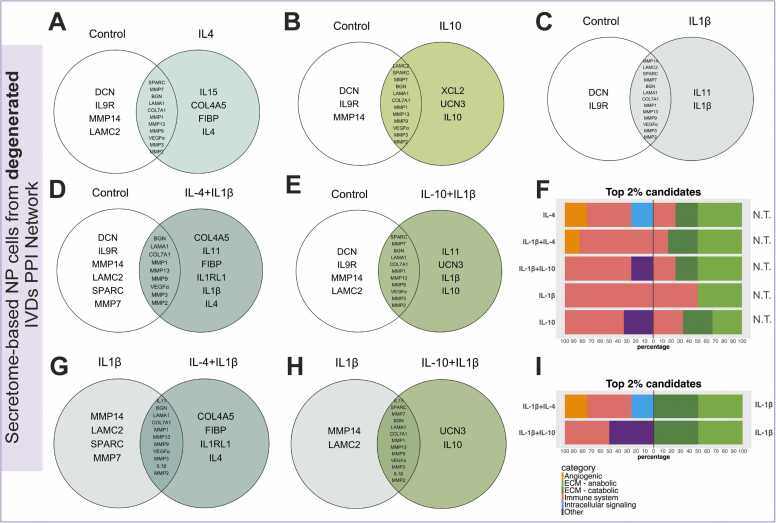
The top 2 % protein candidates uniquely prioritised for 10 ng/ml IL-4, IL-10 and 1 ng/ml IL1β conditions in *in-vitro* degenerated PPI networks developed with secretome data. Venn’s diagram shows the top 2 % of unique protein candidates under (A) IL-4, (B) IL10, (C) IL-1β, (D) IL-4 + IL-1β and (E) IL-10 + IL-1β condition compared to no treatment candidate list. (F) Stacked bar chart of top 2 % protein candidate classification that were uniquely prioritised in IL-4, IL-10, IL-1β, IL-4 + IL1β and IL-10 + IL-1β treatments (left) compared to no treated candidate list (right). Venn’s diagram showing the top 2 % unique protein candidates under (G) IL-4 + IL-1β and (H) IL-10 + IL1β condition compared to IL-1β candidate list. (I) Stacked bar chart of top 2 % protein candidate classification that were uniquely prioritised in IL-4 + IL1β and IL-10 + IL-1Ββ treatments (left) compared to IL-1β candidate list (right). N.T. = No treatment.

The top 2 % ranked candidates from *in-vitro* degenerated PPI networks were selected under IL-4, IL-10 and IL-1β conditions to further evaluate novel vital proteins. Firstly, ECM proteins such as decorin (DCN) and MMP14 together with IL-9R were exclusively identified for the no treatment baseline condition, while IL-15, IL-4, FIBP and COL4A5 were prioritised under the IL-4 condition (Fig. 4A). In addition, X-C motif chemokine ligand 2 (XCL2) and urocortin 3 (UCN3) were exclusively ranked for IL-10 network (Fig. 4B). According to the catabolic IL-1β scenario, IL-11 was prioritised in all the conditions (Figs. 4C, 4D and 4E). Interestingly, IL-1RL1 was uniquely prioritised under combined IL-4 and IL-1β conditions, whereas its baseline incorporated the presence of SPARC and MMP7 proteins as key candidates (Fig. 4D). Furthermore, highlighted candidates from IL-1β and IL-10 case expectedly combined previously prioritised proteins including IL-11 and UCN3 (Fig. 4E). Regarding candidate’s categories, no treated baselines exhibited ECM proteins together with IL9R. In contrast, IL-4 containing conditions showed a variable profile including angiogenic, immune system or intracellular signalling proteins while IL-10 remained consistent with high presence of immune system related proteins (Fig. 4F).

Finally, candidates ranked from combined IL-1β conditions were compared to the IL-1β candidate list baseline. Interestingly, IL-4 condition still prioritised IL-1RL1, COL4A5 and FIBP (Fig. 4G), whereas unicortin remained prioritised for combined IL-10 and IL-1β condition (Fig. 4H). Furthermore, prioritised candidate classification under IL-1β baselines revealed a similar pattern consisting of ECM proteins compared to the no treatment baselines categorization and lacking the immune system related component (Fig. 4I).

### 3.6 IL-10 scenario revealed a diverse prioritised candidate profile on *ex-vivo* PPI networks

**Fig. 5:**
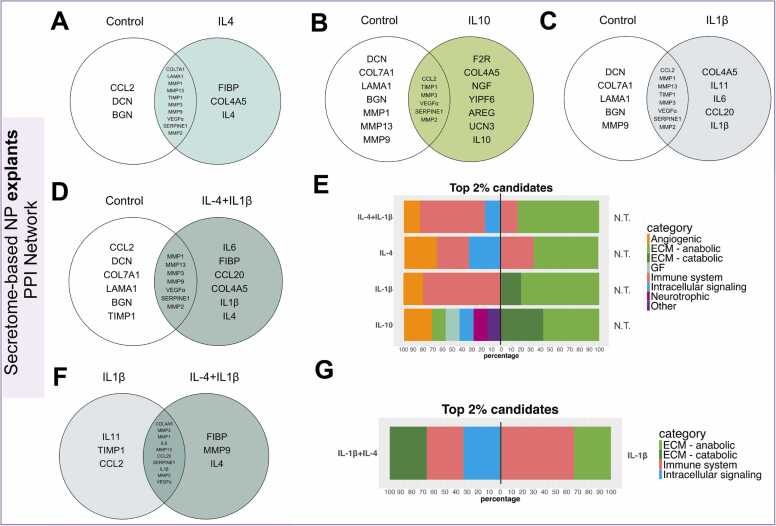
The top 2 % protein candidates uniquely prioritised in 10 ng/ml IL-4, IL-10, and 1 ng/ml IL1β conditions in an *ex-vivo* secretome-based PPI network. Venn’s diagram shows the top 2 % unique protein candidates under (A) IL-4, (B) IL10, (C) IL-1β, and (D) IL-4 + IL-1β condition compared to the no treatment candidate list. (E) Stacked bar chart of top 2 % protein candidate classification that were uniquely prioritised in IL-4, IL-10, IL1β and IL-4 + IL-1β treatments (left) compared to no treated candidate list (right). Venn’s diagram shows the top 2 % unique protein candidates under (G) IL-4 + IL-1β compared to the IL-1β candidate list. (G) Stacked bar chart of top 2 % protein candidate classification that were uniquely prioritised in IL-4 + IL1β condition (left) compared to IL-1β candidate list (right). N.T. = No treatment.

Similar, to the *in-vitro* datasets, *ex-vivo* secretome-based PPI networks were also employed for the top 2 % candidate prioritization. Interestingly, COL4A7 protein was prioritised under all different conditions while DCN and BGN candidates were presented on the no treatment baseline prioritization (Fig. 5). Particularly FIBP, UCN3 and IL-11 were uniquely identified under IL-4, IL-10 and IL-1β conditions, respectively (Figs. 5A, 5B 5 C). Furthermore, IL-6 cytokine and CCL20 chemokine were selected as unique candidates under IL-1β condition (Fig. 5C) whereas proteinase-activated receptor 1 (FR2), neural growth factor (NGF), intracellular transported related YIP1 family member 7 (YIPF7) and AREG were uniquely prioritised under IL-10 condition (Fig. 5B). In terms of IL-4 and IL-1β combined scenario, a combination of IL-4 or IL-1β specific candidates was observed (Fig. 5D). According to the categories, baselines shared a similar profile consisting of ECM related proteins (Fig. 5E). Interestingly, single or combined IL-4 and IL-1β revealed a similar profile including immune system related, angiogenic and intracellular proteins while IL-10 exhibited a multiple categorical pattern consisting of angiogenic, ECM, growth factor, intracellular and neurotrophic proteins (Fig. 5E). Finally, combined IL-4 and IL-1β conditions compared to IL-1β baseline exhibited a particular candidate ranking, highlighting MMP9 and FIBP as the only highly scored proteins (Fig. 5F). Thus, ECM catabolism and intracellular signalling categories were observed for IL-4 and IL-1β conditions (Fig. 5G).

### 3.7 Young and old human patients revealed different candidate prioritization

**Fig. 6:**
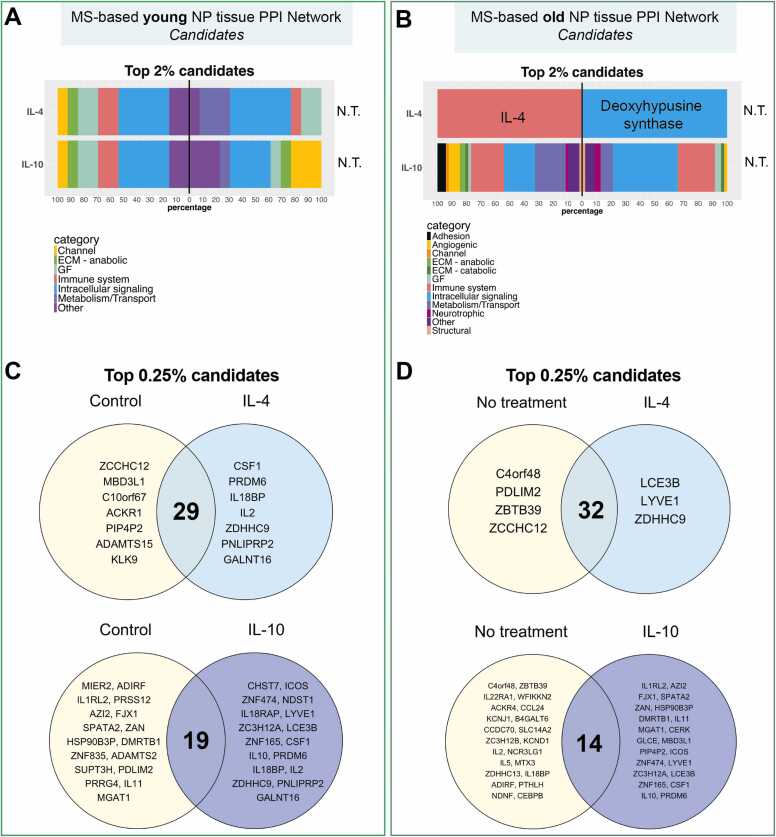
The top 2 % and 0.25 % protein candidates uniquely prioritised under IL-4 and IL-10 scenarios in human NP tissue *MS*-based PPI networks. Stacked bar chart of top 2 % protein candidate classifications that were uniquely prioritised in IL-4 and IL-10 treatments (left) compared to no treated candidate list (right) in (A) young and (B) old human donors. Venn’s diagram showing top 0.25 % unique protein candidates under IL-4 or IL10 condition compared to no treatment candidate list in (C) young and (D) old human donors. N.T. = No treatment; MS= Mass spectrometry.

In addition to the secretome data-based PPI networks, MS-based networks were also developed to further investigate data-driven intracellular pathways. The top 2 % of candidates from MS-based networks for young and old phenotypes were directly analysed due to the higher number of nodes. The young NP tissue network exhibited identical protein prioritization under IL-4 and IL-10 conditions (Table S3). In contrast, prioritised proteins uniquely found in the baseline conditions revealed different profiles, including a higher growth factor component in the baseline condition compared to IL-4, whereas a higher ‘cell channel and transporter’ component was prioritised IL-10 compared baseline (Fig. 6A). Interestingly, candidate prioritization on the old NP tissue network revealed an opposite candidate list for IL-4 and IL-10 conditions. IL-4 was uniquely prioritised for the IL-4 condition, as expected, while dihydropteroate synthase was uniquely prioritised for the no treatment baseline (Fig. 6B). On the other hand, the IL-10 scenario showed multiple candidates related to cell adhesion, angiogenesis, channels, growth factors, immune system, intracellular proteins, metabolic, neurotrophic, and structural categories (Fig. 6B). Similarly, IL-10 respective control group also exhibited a heterogeneous profile, excluding channels and adhesion categories (Fig. 6B).

Finally, the top 0.25 % of candidates were analysed to perform comparisons on approximately 40 candidates, enabling a similar selection to that seen within the secretome-based PPI network top 2 % prioritization. As a result, protein candidates uniquely identified for the IL-4 condition were also listed as unique candidates for IL-10 condition in young NP tissue network (Fig. 6C). Interestingly, the IL-10 condition possessed additional candidates including zinc finger proteins ZNF474 and ZNF165, carbohydrate sulfotransferase 7 (CHST7), inducible T-cell costimulatory (ICOS), bifunctional heparan sulfate N-deacetylase/N-sulfotransferase 1 (NDST1), interleukin-18 receptor accessory protein (IL-18RAP), lymphatic vessel endothelial hyaluronic acid receptor 1 (LYVE1) and late cornified envelope protein 3B (LCE3B) (Fig. 6C). Finally, PPI network built with proteins expressed in the old sample revealed that two out of three prioritised candidates for IL-4 condition (LCE3B and LYVE1) were also found on IL-10 condition candidate list. Interestingly, cGAS and pyroptosis activator palmitoyltransferase ZDHHC9 were uniquely identified under IL-4 condition (Fig. 6D). Additionally, the IL-10 condition revealed additional candidates including IL1RL2, IL-11, 5-azacytidine-induced protein 2 (AZI2), spermatogenesis-associated protein 2 (SPATA2), zonadhesin (ZAN), heat shock protein 90 b3 (HSP90B3P) and doublesex- and mab-3-related transcription factor B1 (DMRTB1) (Fig. 6D).

## 4 Discussion

The PPI network visualization and analysis can provide deeper understanding of biological processes including different pathway interactions. Previously published spaciotemporal IVD-specific human atlas and secretome proteomic studies can be utilized to feed *in-silico* based approaches and further understand the complex interaction between multiple mediators. Particularly, IL-4 and IL-10 have gained attention in the IVD context due to their potential anabolic action [16] and complex pleiotropic nature [32].

MS-based [31] and secretome-based [32] studies were selected due to their secreted messenger and intracellular protein components and utilised for PPI network construction, providing a more comprehensive interaction map of proteins involved IVD - specific biological processes. MS-based prioritised candidate baselines from young and old networks were less interconnected and showed more module-based structure, providing more pathways to explore with well-defined PPI interaction clusters. Nevertheless, donor variability was not considered due to a single young and old patient investigated within the study utilised to build the networks [31]. However, the secretome-based candidate baseline PPI networks offered smaller, highly interconnected, and more representative networks which were developed from three biological replicates in each *in-vitro* and *ex-vivo* scenario. However, due to the limited number of proteins (n = 73) and focus purely on secreted proteins, the resulting PPI networks were not fully connected. Therefore, an enrichment step was performed to generate a fully connected interactome, ensuring successful signal propagation across the nodes.

The top 5 % prioritised candidates on *in-vitro* and *ex-vivo* secretome networks revealed an expected protein candidate profile under no treatment baseline condition, including a higher ECM anabolic or catabolic protein component on trauma networks, while the presence of ‘immune system’ related pro-catabolic cytokines and chemokines was enhanced on the degenerated network, as previously reported in experimental IVD degeneration studies [9], [38]. Thus, a consistent baseline of prioritized candidates was observed according to the NP cell phenotype. Furthermore, integrin-mediated mechanotransduction pathways were exclusively prioritised in the degenerated PPI network, suggesting a potential involvement of integrin β3 during degeneration, which has been previously described in human IVDs [44]. Interestingly, prior studies in integrin-mediated mechanotransduction revealed different pathways in non-degenerated and degenerated NP cells, whereby whilst arginine-glycine-aspartic acid (RGD) integrins (possibly α5β1) were involved in mechanotransduction pathways in NP cells derived from non-degenerate discs this was disrupted in those cells derived from degenerate discs [45].

**Fig. 7:**
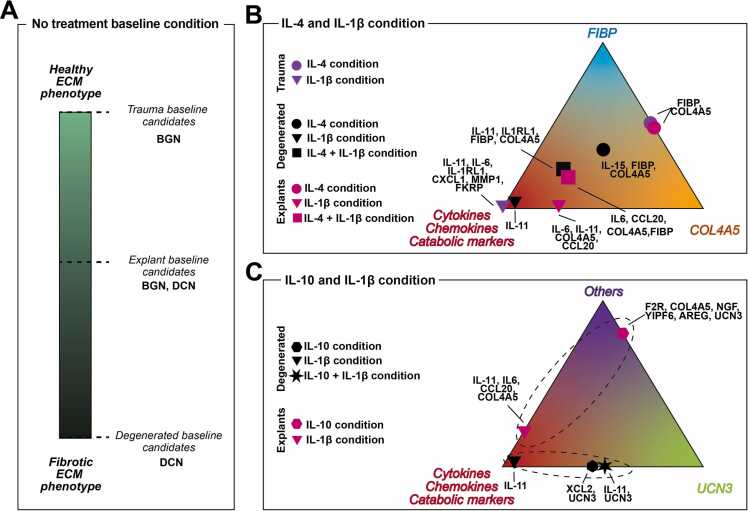
Conceptual overview of no treatment, IL-4, IL-10, and I IL-1β candidate prioritisation. (A) No treatment baseline candidates according to ECM proteoglycan components. (B) Prioritised candidates under IL-4 and IL-1β conditions according to culture system and category. (C) Prioritised candidates under IL-10 and IL-1β conditions according to culture system and category. A dashed circle indicates a culture system-dependent effect. AREG: Amphiregulin; BGN: Biglycan; CCL20: C-C Motif Chemokine Ligand 20; COL4A5: Collagen Type IV Alpha 5 Chain; CXCL1: C-X-C Motif Chemokine Ligand 1; DCN: Decorin; F2R: Coagulation Factor II Receptor; FIBP: Fatty Acid-Binding Protein; FKRP: Fukutin Related Protein; IL10: Interleukin 10; IL11: Interleukin 11; IL1RL1: Interleukin 1 Receptor-Like 1; IL15: Interleukin 15; IL6: Interleukin 6; MMP1: Matrix Metalloproteinase 1; NGF: Nerve Growth Factor; UCN3: Urocortin 3; YIPF6: Yippee-Like 6; XCL2: C-X3-C Motif Chemokine Ligand 2.

A smaller percentage of candidate ranking can uncover more detailed protein-protein relationships, emphasizing key protein players under different conditions. Particularly, the top 2 % of candidates on secretome-based networks revealed different prioritised candidate baselines between trauma, degenerated, and explant networks. For example, biglycan, whose absence has been reported to accelerate IVD degeneration [46], was prioritised in the trauma PPI network under no treatment baseline condition. On the other hand, DCN was highlighted in the degenerated PPI network baseline. Previous studies have identified the presence of biglycan and decorin proteoglycans in human and ovine IVD tissue [46], [47], whereby decorin is expressed by fibroblastic-like cells and highly present on fibrillar NP areas whilst biglycan is found within healthy NP tissue [47], [48]. In addition, *ex-vivo* candidate prioritisation revealed both small proteoglycans as candidates due to a possible intermediate healthy and degenerated phenotype NP explants might possess. Hence, *in-vitro* and *ex-vivo* secretome-based PPI networks prioritization reflected NP cell ECM phenotypic features and mimicked the experimental outcomes (Fig. 7A). Similarly, our experiments were able to mimic the IL-1β mediated catabolic effect both ex-vivo and in-vitro systems, prioritising IL-6 family members including IL-6 and IL-11 respectively, which have been described following IL-1β stimulation [9], [49], [50] (Fig. 7B). Additionally, IL-9R was consistently and exclusively identified in the degenerated PPI networks, being IL-9 cytokine previously reported to promote pro-catabolic and inflammatory TNF secretion in human IVD tissue [51]. Thus, the IL-9/IL-9R signalling pathway might be key in human NP cells from degenerated IVDs.

On the other hand, our findings could propose novel candidates (*underlined*) from trauma and degenerated PPI under IL-4 and IL-10 conditions. Regarding IL-4, collagen type IV (COL4A5) was prioritized under IL-4 containing conditions in *in-vitro* and *ex-vivo* experiments, suggesting a potential link between collagen type IV and IL-4 in the IVD. Prior studies have described collagen IV presence on the pericellular matrix of NP cells [52], [53], [54] and suggested that collagen IV might play a role in the adaptative immune response to altered microenvironments in musculoskeletal degenerative diseases [53]. Fibroblast growth factor intracellular binding protein (FIBP) was also prioritized in IL-4 conditions in both in-vitro and ex-vivo PPI networks. Interestingly, FIBP has been reported to interact with the STAT3 signalling pathway [55], which also can be activated by IL-4 cytokine [56]. In contrast, IL-15, expression of which has been shown to correlation with age and IVD degeneration grade and has been attributed to a possible pathological role in disc diseases [57], was also uniquely prioritised under IL-4 condition exclusively on degenerated PPI network, suggesting a phenotype-dependent candidate prioritisation (Fig. 7B). Soluble IL-1 receptor-like 1 (IL-1RL1) was prioritised in the degenerated PPI network following IL-4 and IL-1β conditions, enhancing IL-1β catabolic response (Fig. 7B). Notably, in-vitro IL-IRL1 secretion is significantly upregulated exclusively in IVD degeneration patients [32] and contributes to radicular pain in mice models [58]. Thus, a proposed pleiotropic IL-4 effect in degenerated IVD context previously reported [32] was also observed *in-silico*, highlighting our model’s ability to mimic experimental results. For the IL-10 scenario, the urocortin 3 candidate was the top 2 % listed candidate in in-vitro and ex-vivo secretome-based PPI networks. Noteworthy, urocortin has been reported as a chondroprotective factor [50] for NP-induced cell apoptosis [59]. In addition, NGF was additionally listed as a critical protein candidate in the explant PPI network, as previously reported in the experimental secretome study [32]. Hence, in this study has developed PPI networks to replicate experimental findings under IL-4 and IL-10 stimulations and predict potential candidates. Interestingly, IL-10-containing conditions showed a culture-system dependent categorisation, in which the principal candidates from the in-vitro scenario were urocortin 3 and cytokine, chemokine and catabolism-oriented while in the explant context, other factors were highlighted (Fig. 7C).

Finally, MS-based, more extensive PPI networks were also utilised for protein candidate prediction. The top 2 % prioritised candidates PPI network built with expressed proteins within discs from young individuals for IL-4 condition were also found under IL-10 condition, indicating a trivial IL-4 role on a young phenotype. In addition, the old PPI network predicted similar protein candidate prioritisation between IL-4 and no treatment baseline. Regarding novel candidates, palmitoyltransferase (ZDHHC9) protein was ranked in the top 0.25 % candidates uniquely for IL-4 condition, indicating the importance of this protein in IL-4 pathway. Moreover, ZDHHC9 protein can catalyze direct pore formation during pyroptosis [60] and additionally activate cGAS pathway [61], key processes involved in two current topics in the IVD field: including the potential role of bacterial microbiome and its influence on pyroptosis [62], [63] and senescence [64], [65]. In contrast, the top 0.25 % prioritised candidates under the IL-10 condition revealed 22 potentially regulated proteins, including interleukins, transcription factors and intracellular signalling proteins.

## 5 Conclusion

In conclusion, signal propagation methods on PPI networks built with proteins measured in an experimental setting can behave like in-vitro or *ex-vivo* experiments and reliably prioritise potential protein candidates involved in the ECM structure, cell signalling and catabolic responses. Network-based models strongly rely on high throughput technologies that can generate predictions based on existing data and theoretical frameworks that may not fully capture the complexity of biological systems and must be experimentally validated. *In-silico* models can prioritise and predict the catabolic IL-1β response of NP cells and revealed IL4 pleiotropic effects, prioritising IL-15 on degenerated phenotypes or highlighting IL-1RL1 in the presence of IL-1β. Additionally, new insights on potential candidates for IL-4 or IL-10 investigations on degenerated IVDs were obtained, including collagen type IV, FIBP, as well as UCN3 and NGF, respectively. Furthermore, IL1RL1 roles in the presence of IL-1β and IL-4 was proposed. The nature of the input data must be considered due to the lack of experimental intracellular protein information on the secretome-based approach. MS-based PPI networks were built with a large-scale data set including intracellular protein links. Additionally, potential biases in the input data must be considered. Interactions are often reported more frequently for well-known or extensively studied proteins, particularly those of medical relevance. Conversely, less characterized proteins may lack documented interaction data. Furthermore, a key limitation is the lack of biological variability as they were based from low numbers of individual profiles. Combining multiple proteomic experimental approaches for *in-silico* validation is required to fully understand the complex interaction between many proteins involved in a multifactorial disease such as IVD degeneration.

## Author contributions

**FG, BO, JP, CLM**, and **PB-L** conceptualised the study; **FG** developed a PPI network occasionally assisted by **PB-L; FG** ran the GUILD algorithm (previously developed by BO) on PPI networks; **PB-L** was responsible for data acquisition, analysis, interpretation, and visualisation assisted by **FG**; **FG** and **PB-L** wrote the original draft; **BG, BO, JP**, and **CLM** reviewed and edited the manuscript and sourced funding.

## CRediT authorship contribution statement

**Bermudez-Lekerika Paola:** Writing – original draft, Visualization, Validation, Methodology, Investigation, Formal analysis, Data curation, Conceptualization. **Gualdi Francesco:** Writing – original draft, Software, Resources, Methodology, Investigation, Formal analysis, Data curation, Conceptualization. **Le Maitre Christine:** Writing – review & editing, Supervision, Project administration, Investigation, Funding acquisition, Data curation, Conceptualization. **Piñero Janet:** Writing – review & editing, Supervision, Software, Resources, Data curation, Conceptualization. **Oliva Baldomero:** Writing – review & editing, Supervision, Project administration, Methodology, Investigation, Formal analysis, Data curation, Conceptualization. **Gantenbein Benjamin:** Writing – review & editing, Supervision, Investigation, Funding acquisition, Conceptualization.

## Funding sources

This work was supported by the Marie Skłodowska Curie International Training Network (ITN) “disc4all” (https://disc4all.upf.edu, accessed on 9 April 2024) by the grant agreement #955735 (https://cordis.europa.eu/project/id/955735, accessed on 9 April 2025).

## Declaration of Competing Interest

The authors declare that they have no known competing financial interests or personal relationships that could have appeared to influence the work reported in this paper. Benjamin Gantenbein and Christine Le Maître report financial support for this work was provided by Marie Skłodowska Curie International Training Network (ITN) “disc4all” (https://disc4all.upf.edu, accessed on 7 Jan 2025) grant agreement #955735. If there are other authors, they declare that they have no known competing financial interests or personal relationships that could have appeared to influence the work reported in this paper.

## Footnotes

Appendix A Supplementary data associated with this article can be found in the online version at doi:10.1016/j.csbj.2025.04.015.
