# Supplementary material for: In-silico proteomic analysis of the role of IL-4 and IL-10 in IVD degeneration: Protein-protein interaction networks for candidate prioritisation
Source: Comput Struct Biotechnol J. 2025 Apr 14;27:1600–13. doi: 10.1016/j.csbj.2025.04.015 (PMC12033940; doi:10.1016/j.csbj.2025.04.015)
Supplement: Supplementary file 1 — Supplementary material [file mmc1.docx]

**Table S1:**  Top 5% prioritized protein candidates in secretome-based PPI networks

| **Data set** | ***Top 5% Prioritized Protein Candidates*** | | | | | | | | | | | | | | | |
| --- | --- | --- | --- | --- | --- | --- | --- | --- | --- | --- | --- | --- | --- | --- | --- | --- |
|  | **No treatment** | **IL-4** | **No treatment** | **IL-10** | **No treatment** | **IL-1β** | **No treatment** | **IL-1β +**  **IL-4** | **No treatment** | **IL-1β + IL-10** | **IL-1β** | | **IL-1β + IL-4** | **IL-1β** | | **IL-1β + IL-10** |
| *Secretome proteomics:*  *NP Explants*  *(Ex vivo)* | FSTL1  HAPLN1  CTSG  AREG  COL2A1  FBXO2  CSF3  THBS1  LAMC2  VCAN  F2R | YIPF6  SDC1  JAGN1  IL13  MYOC  CREB3L1  ASGR2  TNFRSF11B  RETN  FIBP  **IL4** | FSTL1  CTSG  COL2A1  FBXO2  CSF3  THBS1  LAMC2  VCAN  COL4A3  HLA-DQA1  MMP14  SPARC  MMP7  DCN  BGN | SRGN  IL11  FKRP  XCL2  EGF  TNFRSF11B  NMU  MYOC  TIMP3  SNTG1  JAGN1  RETN  YIPF6  UCN3  **IL10** | FSTL1  CTSG  AREG  COL2A1  FBXO2  CSF3  THBS1  LAMC2  VCAN  HLA-DQA1  MMP14  SPARC  MMP7  DCN | IL1A  VWDE  XCL2  BCAN  IL1RAP  CCL21  CHRNA5  JAGN1  CXCL10  CXCL1  CCL7  IL11  IL6  **IL1β** | FSTL1  HAPLN1  CTSG  AREG  COL2A1  FBXO2  CSF3  THBS1  LAMC2  VCAN  F2R  SPOCK1  HLA-DQA1  MMP14  MMP7 | CREB3L1  SDC1  CXCL1  MYOC  YIPF6  ASGR2  IL11  IL13  JAGN1  TNFRSF11B  RETN  IL6  FIBP  **IL1β**  **IL4** | N/A | N/A | IL1A  VWDE  XCL2  BCAN  IL1RAP  F2R  CCL21  HAPLN1  SPOCK1  CHRNA5  CXCL10  CCL7 | | SPARC  CREB3L1  SDC1  DCN  MYOC  YIPF6  ASGR2  IL13  TNFRSF11B  RETN  FIBP  **IL4** | N/A | | N/A |
| *Unique/total*  *candidates* | 11/34 | 11/34 | 15/34 | 15/34 | 14/34 | 14/34 | 15/34 | 15/34 | - | - | 12/22 | | 12/22 | - | | - |
| *Secretome proteomics: NP cells from degenerated IVDs*  *(In vitro)* | ACKR1  PRG2  SERPINE1  CSF3  FBXO2  EGF  ITGB3  TIMP1 | IL7  KLK2  IL2  IL13  RETN  ASGR2  FIBP  **IL4** | TNFRSF11B  ACKR1  PRG2  SERPINE1  CSF3  FBXO2  CREB3L1  EGF | NMU  KLK2  IL22  AREG  RETN  XCL2  UCN3  **IL10** | TNFRSF11B  PRG2  SERPINE1  MYOC  EGF  ITGB3 | CCL20  OSM  CCL2  IL6  IL11  **IL1β** | TNFRSF11B  ACKR1  PRG2  SERPINE1  CSF3  FBXO2  EGF  ITGB3  LRP1  VCAN  COL2A1  HLA-DQA1 | KLK2  IL7  IL2  IL13  ASGR2  RETN  IL6  IL11  FIBP  IL1RL1  **IL1β**  **IL4** | TNFRSF11B  ACKR1  PRG2  SERPINE1  CSF3  FBXO2  CREB3L1  EGF  ITGB3  TIMP1  LRP1 | IL4  IL22  KLK2  IL6  AREG  XCL2  RETN  IL11  UCN3  **IL1β**  **IL10** | FBXO2  CCL20  OSM  ACKR1  LRP1  CCL2  CSF3  COL2A1  VCAN  HLA-DQA1 | | MYOC  KLK2  IL7  IL2  IL13  ASGR2  RETN  FIBP  IL1RL1  **IL4** | FBXO2  CCL20  OSM  CREB3L1  ACKR1  LRP1  CCL2  CSF3  TIMP1 | | IL4  IL22  KLK2  MYOC  AREG  XCL2  RETN  UCN3  **IL10** |
| *Unique/total candidates* | 8/39 | 8/39 | 8/39 | 8/39 | 6/39 | 6/39 | 12/39 | 12/39 | 11/39 | 11/39 | 10/39 | | 10/39 | 9/39 | | 9/39 |
| *Secretome proteomics: NP cells from trauma IVDs*  *(In vitro)* | SERPINA3  FSTL1  THBS1  LAMC2  CSF3  SNTG1  TIMP3  SRGN | LRP5  CXCL16  IL7  IL13  IL15  RETN  FIBP  **IL4** | N/A | N/A | SERPINA3  FSTL1  THBS1  LAMC2  CSF3  SNTG1  TNFRSF11B  SRGN  SDC1  EGF  COL2A1  MMP14  MMP7  DCN  F2R | ATP8B2  CD53  OSM  RETN  CASP1  PPP1R12C  LIF  CTPS2  CXCL12  COL4A3  CXCL1  IL11  **IL1β**  IL6  IL1RL1 | N/A | N/A | N/A | N/A | N/A | | N/A | N/A | | N/A |
| *Unique/total*  *candidates* | 8/39 | 8/39 | - | - | 15/39 | 15/39 | - | - | - | - | - | - | | | - | - |
