# Supplementary material for: In-silico proteomic analysis of the role of IL-4 and IL-10 in IVD degeneration: Protein-protein interaction networks for candidate prioritisation
Source: Comput Struct Biotechnol J. 2025 Apr 14;27:1600–13. doi: 10.1016/j.csbj.2025.04.015 (PMC12033940; doi:10.1016/j.csbj.2025.04.015)
Supplement: Supplementary file 2 — Supplementary material [file mmc2.docx]

**Table S2**: Top 2% prioritized candidates in secretome-based PPI networks

| **Condition** | | **Prioritized categories** | **Unique Proteins** |
| --- | --- | --- | --- |
| **Trauma PPI Network** | ***No treatment***  ***Baseline*** | ECM related anabolic and catabolic proteins | BGN, SPACK, F2R, |
|  | ***IL-4 condition*** | Angiogenic and intracellular signaling proteins | COL4A5, FIBP, IL4 |
|  | ***IL-1β condition*** | Immune system related and catabolic proteins | IL-11, IL-6, IL1RL1, CXCL1, MMP1, FKRP, |
| **Degenerated PPI Network** | ***No treatment***  ***Baseline*** | ECM and immune system related proteins | DCN, ILR9 |
|  | ***IL-4 condition*** | Immune system related proteins and angiogenic proteins | IL-15, FIBP, COL4A5, IL-4 |
|  | ***IL-10 condition*** | Immune system proteins | XCL2, UCN3, IL10 |
|  | ***IL-1β condition*** | Immune system proteins and growth factor receptors | IL-11 IL-1β |
|  | ***IL-1β + IL-4 condition*** | Principally immune system related proteins | IL-11, IL1RL1,FIBP, COL4A5, IL-4, IL-1β |
|  | ***IL-1β + IL-10 condition*** | Principally immune system related proteins | IL-11, UCN3, IL1β, IL10 |
| **Explants PPI Network** | ***No treatment***  ***Baseline*** | Mainly ECM related anabolic proteins | DCN, BGN |
|  | ***IL-4 containing conditions*** | Intracellular signalling, angiogenic and immune system related proteins | COL4A5, FIBP, IL4 |
|  | ***IL-1β condition*** | Mainly immune system proteins | IL6, IL-11, COL4A5, CCL20, IL1β |
|  | ***IL-10 condition*** | Heterogeneous categorization | F2R, COL4A5, NGF, YIPF6, AREG, UCN3,IL-10 |
|  | ***IL-1β + IL-4 condition*** | Principally immune system related proteins | IL6, COL4A5, CCL20, FIBP, IL1β, IL4 |

AREG: Amphiregulin; BGN: Biglycan; CCL20: C-C Motif Chemokine Ligand 20; COL4A5: Collagen Type IV Alpha 5; DCN: Decorin; F2R: Coagulation Factor II Receptor; FIBP:FGF1-Binding Protein; FKRP: Fukutin Related Protein; IL10: Interleukin 10; IL11: Interleukin 11; IL1β: Interleukin 1 Beta; IL4: Interleukin 4; IL6: Interleukin 6; IL15: Interleukin 15; ILR9: Interleukin 9 Receptor; IL1RL1: Interleukin 1 Receptor-Like 1; MMP1: Matrix Metalloproteinase 1; NGF: Nerve Growth Factor; SPACK: SPARC-Related Modular Calcium-Binding Protein; UCN3: Urocortin 3; YIPF6: *Yip1 Domain Family Member 6*; XCL2: X-C Motif Chemokine Ligand 2.
