# Supplementary material for: In-silico proteomic analysis of the role of IL-4 and IL-10 in IVD degeneration: Protein-protein interaction networks for candidate prioritisation
Source: Comput Struct Biotechnol J. 2025 Apr 14;27:1600–13. doi: 10.1016/j.csbj.2025.04.015 (PMC12033940; doi:10.1016/j.csbj.2025.04.015)
Supplement: Supplementary file 3 — Supplementary material [file mmc3.docx]

**Table S3**: Top 2% prioritized unique proteins candidates from MS-based PPI networks

| **Data set** | ***Top 2 % Prioritized Unique Protein Candidates*** | | | | | | |
| --- | --- | --- | --- | --- | --- | --- | --- |
|  | **No treatment** | **IL-4** | **No treatment** | | **IL-10** | |  |
| *MS proteomics:*  *NP tissue from Young IVDs* | PSG9  TNFSF9  SEMA3F  LCE2C  LARP4  HOOK2  TGFBRAP1  RNF19B  KCTD5  ORC5  SMPD3  BRWD3  LGALS14 | PRSS50  KRTAP4-11  IL12A  SPARCL1  KCND2  JAKMIP1  LCE3D  BMPR1B  PPP1R16A  PGF  TAB3  SMU1  I**L4** | SERPINB7  FABP4  PDF  FHL5  TRPM3  PIK3CG  IGFBP2  TMEM263  ABHD15  MYOG  SLC35C2  GRIN2B  AXIN2 | | PRSS50  KRTAP4-11  IL12A  SPARCL1  KCND2  JAKMIP1  LCE3D  BMPR1B  PPP1R16A  PGF  TAB3  SMU1  **IL10** | |  |
| *N/total candidates=* | 13/292 | 13/292 | 13/292 | | 13/292 | |  |
| *MS proteomics:*  *NP tissue from Old IVDs* | DHPS | **IL4** | DHPS  GALNT7  NRBP2  PYCARD  SORT1  CXCR2  LTBR  LINGO1  SLC22A2  ZNF581  PDIK1L  INTS5  NSMF  GALNT6  ZNF572  HSF2BP  USHBP1  ZFP36  CASP4  SOS1  NTMT1  NT5DC3  NDNF | BCL10  MLKL  SMU1  CSGALNACT2  CXCL11  TBKBP1  FAM120C  ZNF526  LRRC32  CCL13  CCNH  CXCL10  EPHB2  PRR13  PPBP  BDKRB1  SOHLH1  CCL21  CXCL14  CCDC33  ADAM15  BMP3  MED15  CDC45 | SMAD7  NMT2  SERPINB7  FABP4  PDF  IGFBP2  SLC35C2  GRIN2B  AXIN2  NCALD  ZNF747  FAM124A  SLC30A4  SLC22A1  TNFSF9  SEMA3F  RNF19B  KCTD5  SMPD3  LGALS14  PRSS50  SPARCL1  NR2F2  NTN1  SRGN  CCL20 | POMGNT1  CLDN7  IGSF1  IL1R2  WRNIP1  FFAR2  VGF  SCG2  ARSB  IL20RB  BCAS3  CST5  FNDC9  PF4  AP2S1  IL1RAP  PIK3R5  LRRC24  LOXL1  MMP15  CCL16  GALNT16  TLR3  IL11  MGAT1  **IL10** |  |
| *N/total candidates=* | 1/287 | 1/287 | 47/292 | | 52/292 | |  |
